# Medical malpractice related to dialysis and vascular access: An analysis of lawsuit judgements in South Korea

**DOI:** 10.1371/journal.pone.0255020

**Published:** 2021-08-05

**Authors:** Ji Eun Kim, Shin Young Ahn, Soo Ick Cho, Young Joo Kwon, SuHwan Shin, Gang-Jee Ko

**Affiliations:** 1 Department of Internal Medicine, Korea University Guro Hospital, Seoul, Korea; 2 Department of Dermatology, Seoul National University Hospital, Seoul, Korea; 3 Blue Urology Clinic, Seoul, Korea; 4 Department of Medical Law and Ethics, Graduate School, Yonsei University, Seoul, Korea; University of KwaZulu-Natal, SOUTH AFRICA

## Abstract

**Background:**

Hemodialysis is a life-saving renal replacement treatment for patients with chronic kidney disease, but various complications occur during hemodialysis and associated procedures. This study was conducted to analyze the specific characteristics of hemodialysis-related complications and malpractice that have led to legal disputes.

**Methods:**

Judgments from cases litigated between 1991 and 2019 due to complications related to hemodialysis or vascular access were analyzed using the database of the Korean Supreme Court Judgment System.

**Results:**

Of 32 dialysis-related litigation cases, 14 cases were dismissed and malpractice was recognized in 18 cases. Among all cases and those in which malpractice was recognized, the most common clinical complication was associated with central venous catheter (CVC) insertion (25.0% and 42.9%, respectively). In 22 of 32 (68.8%) cases, complications occurred before or after (not during) dialysis, and performance error was the most common clinical error leading to legal disputes (58.3%). Complications resulted in death in 59.4% of cases, and CVC-related complications were associated with the largest proportion (63.2%) of deaths.

**Conclusions:**

Hemodialysis was implicated in various medical disputes, and CVC-related complications were the most common and serious adverse events. Clinicians’ awareness of the incidence and severity of possible complications of hemodialysis procedures should be increased.

## Introduction

Hemodialysis is a life-saving treatment that has been administered to patients with chronic kidney disease for several decades [1]. Although hemodialysis has evolved into a relatively safe procedure, various complications can occur, and some have serious consequences [2–6].

Patients with chronic kidney disease who require hemodialysis are at risk of various complications associated with uremia [7]. Although hemodialysis is believed to improve most uremic symptoms [8, 9], the dialysis procedure may be implicated in the incidence of various complications [4–6]. In particular, many complications are associated with procedures undertaken to prepare for hemodialysis, such as central venous catheter (CVC) placement and surgery performed to obtain vascular access [10–12]. Although the uremic milieu confers vulnerability in many cases in which accidents occur, medical team negligence triggers and exacerbates many hemodialysis -related complications [13, 14] and should be prevented.

This study involved the analysis of judgments in cases litigated in the Korean court system with the goal of proving medical malpractice related to hemodialysis and associated procedures. An analysis of legal judgments of medical malpractice may aid the identification and prevention of rare, but serious, hemodialysis complications generating malpractice claims. It may also help to raise awareness of the possibility of hemodialysis -related malpractice and promote greater attention to the potential occurrence of rare, but important, complications throughout the hemodialysis process.

## Materials and methods

We analyzed publicly accessible legal judgments in the database of the judicial system of the Supreme Court of Korea. This database contains judgments from civil proceedings tried from the district court to the Supreme Court level [15, 16]. Identifiable information has been removed from the data. All relevant medical malpractice cases for which sentences were given between January 1, 1991, and December 31, 2019, were retrieved using the search terms “hemodialysis,” “vascular access,” “arteriovenous fistula,” and “arteriovenous graft.” We excluded duplicate cases and those unrelated to hemodialysis. The Institutional Review Board of Korea University Hospital approved this study (no. 2020GR0174) and waived the requirement of informed consent.

Each judgment record contained a detailed narrative of the case, the plaintiff’s malpractice claim, and the court’s decision regarding medical malpractice. Two board-certified nephrologists reviewed these records and collected the following information: year of event, patient age and sex, underlying medical diseases, hemodialysis indication and duration, symptoms before or after hemodialysis /catheter insertion/arteriovenous fistula (AVF) creation, and complication type and severity. Data on the detailed plaintiff claims, court opinions, and final monetary amounts awarded were also collected. The plaintiffs’ allegations in relation to hemodialysis procedures were classified as violations of the duty of care and violations of the duty to explain.

Descriptive statistics were calculated using R software (version 3.5.0; R Foundation for Statistical Computing, Vienna, Austria). Categorical data are described as percentages, and continuous data are described as medians (interquartile ranges [IQRs]).

## Results

**Fig 1:**
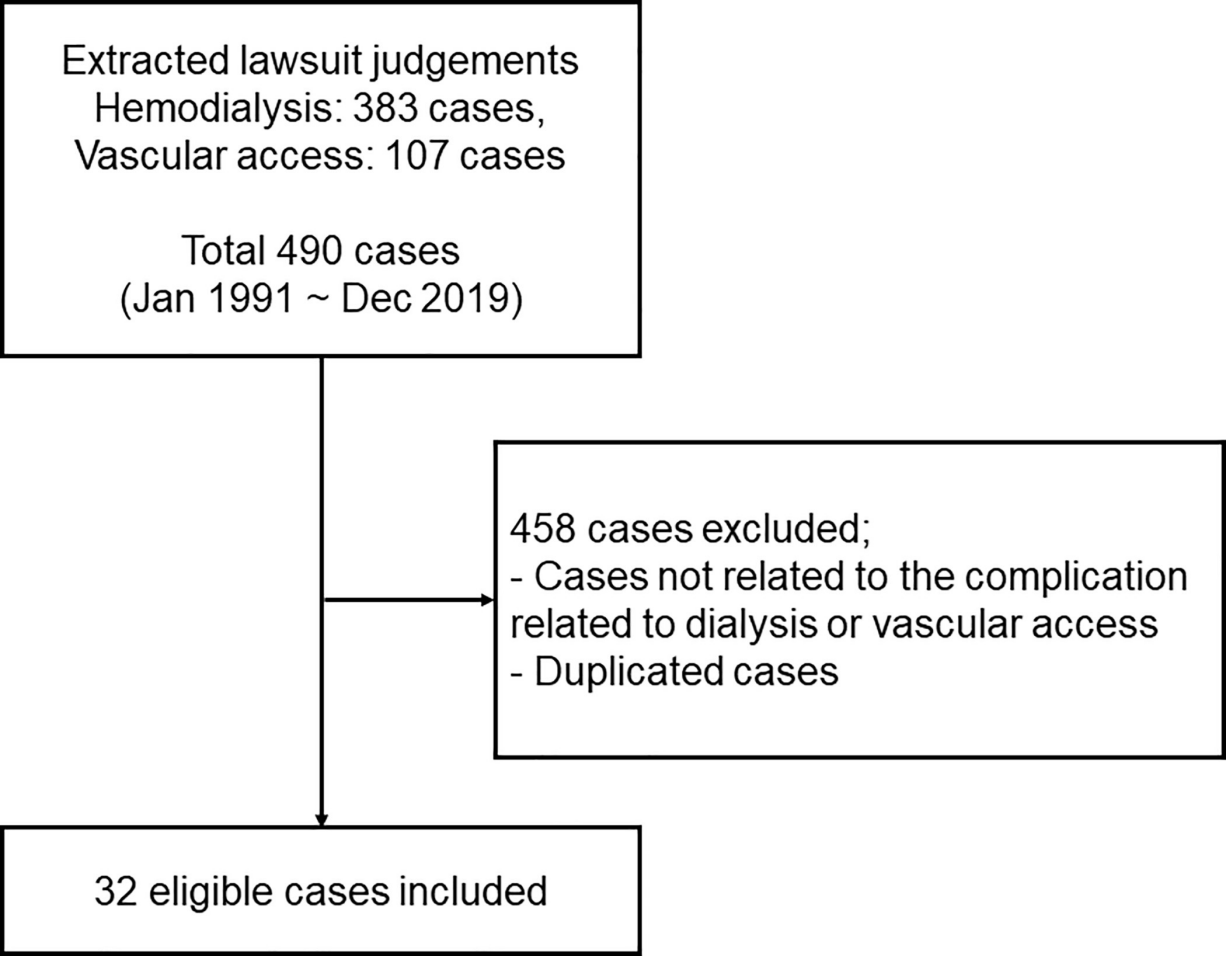
Flowchart of judgement selection based on the inclusion and exclusion criteria.

Of the 490 potentially eligible case records obtained by keyword search, 458 unrelated or duplicate records were excluded and 32 records of cases associated with hemodialysis treatment were retained. Cases associated with vascular access were included in the final analysis (Fig 1). hemodialysis was performed to treat acute kidney injury in six (18.8%) cases and to treat chronic kidney disease in the other cases. In 12 (37.5%) of the 32 cases, complications occurred within 1 year after hemodialysis initiation. Death occurred in 19 (59.4%) cases and permanent disability occurred in nine (28.1%) cases. The demographic and clinical characteristics of the patients involved in the cases are presented in Table 1.

**Table 1:** Clinical characteristics of the litigation cases.

Characteristics	N = 32
Sex	
Male	13 (40.6%)
Female	8 (25.0%)
Not described	11 (34.4%)
Age, years	
< 40	4 (12.5%)
≥ 40	6 (18.8%)
Not described	22 (68.8%)
Date of complication	
`90s	5 (15.6%)
`00s	12 (37.5%)
`10s	15 (46.9%)
Dialysis vintage	
Not described	7 (21.9%)
< 1 month	7 (21.9%)
≤ 1 year	5 (15.6%)
1 < and ≤ 5 year	7 (21.9%)
5 < year	6 (18.8%)
Characteristics of kidney disease	
Acute kidney injury	6 (18.8%)
Chronic kidney disease	26 (81.3%)
Underlying disease	
Diabetes mellitus	7 (21.9%)
Hypertension	6 (18.8%)
Hematologic disease	2 (6.3%)
Cardiovascular disease	2 (6.3%)
Liver disease	2 (6.3%)
Neurologic disease	2 (6.3%)
Pulmonary disease	1 (3.1%)
Infectious disease	1 (3.1%)
Not described	18 (56.3%)
Grave injury	
Death	19 (59.4%)
Disability	9 (28.1%)

Hospitals were the defendants in 24 cases, and doctors and nurses were claimed as the defendants in 10 and 3 cases, respectively. The median claim amount was $72,506 (IQR, $27,144–262,977). Damages were awarded to the plaintiffs in 18 (56.3%) cases, and the median amount was $21,667 (IQR, $9,583–73,807). The overall characteristics of the lawsuits are presented in S1 Table.

The most common clinical causes of litigation were vascular access–related complications. Eighteen (56.3%) cases were associated with the management of vascular access (CVC insertion [*n* = 8], AV access [*n* = 7], AV access cannulation [*n* = 2], and AV access thrombosis [*n* = 1]). Twenty-four cases involved performance error and five cases involved diagnostic error. Improper complication management was alleged in 12 cases.

Defendant negligence was recognized in 18 (56.3%) of the 32 cases, the majority (*n* = 11 [61.1%]) of which involved vascular access–associated complications. Six of these cases were associated with CVC insertion, which was the most common cause of malpractice claims, and negligence in instruction and explanation of this procedure was claimed in three of these six cases. The clinical and legal negligence characteristics of the cases are shown in Table 2.

**Table 2:** Classification of reason for litigation.

	Complications related to disputed cases		Allegation of plaintiff	Recognition by Court
			N = 32	N = 18
By clinical characteristics	Vascular access related	CVC insertion	8	6 (75.0%)
		AV access formation	7	4 (57.1%)
		AV access cannulation	2	1 (50.0%)
		AV access thrombosis	1	0 (0%)
	Infection		4	2 (50.0%)
	Patient monitoring		3	2 (66.7%)
	Dialysis circuit clot		2	0 (0%)
	Dialysis delay		1	1 (100%)
	Others		4	2 (50.0%)
By negligence categories	Diagnosis error		5	3 (60.0%)
	Performance error		24	14 (58.3%)
	Improper management for complication		12	7 (58.3%)
	Lack of informed consent		2	2 (100%)
	Improper instruction and/or explanation		7	3 (42.9%)

Abbreviations: CVC, central venous catheter; AV, arteriovenous

**Fig 2:**
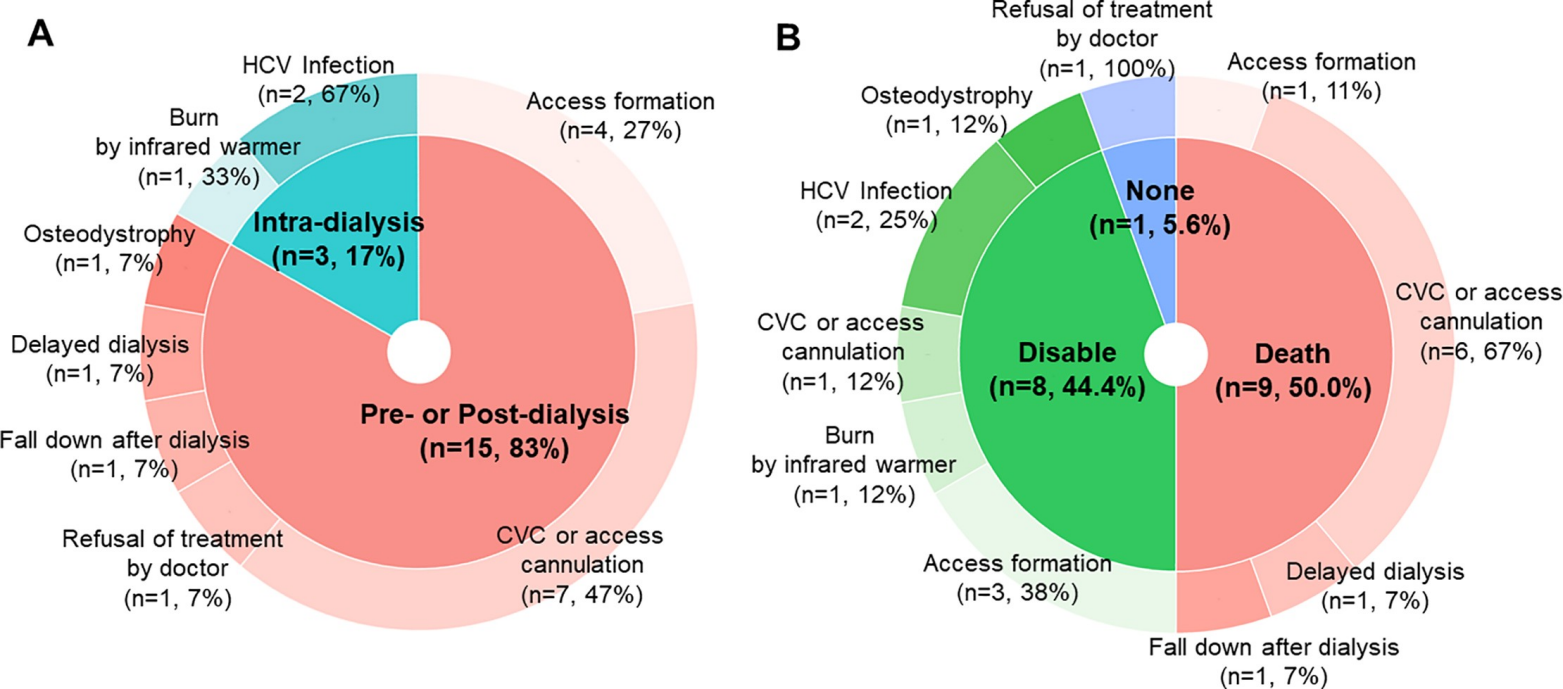
Characteristics of malpractice recognized by the court. A. Temporal characteristics according to clinical characteristics of malpractice. B. Grave outcomes according to clinical characteristics of malpractices. The percentages in pie chart represent proportions in whole malpractices and the percentages in donut chart represent sub-proportions in each pie chart.

Complications occurred during dialysis in 10 (31.3%) cases, and before (*n* = 14) or after (*n* = 8) dialysis in 22 (68.8%) cases. Four complications that occurred during dialysis were attributable to infection (with hepatitis C virus [*n* = 2], *Staphylococcus aureus* [*n* = 1], and an unidentified pathogen [*n* = 1]). In contrast, most (*n* = 18 [81.8%]) complications that occurred before or after dialysis were associated with CVC implantation for vascular access. The court recognized negligence in more cases involving complications that occurred before or after dialysis (*n* = 15 [68.2%]) than in those involving complications that occurred during dialysis (*n =* 3 [33.3%]). Moreover, negligence was recognized in 11 (66.7%) cases associated with vascular access. The detailed clinical characteristics of the malpractice cases, classified according to complication timing, are presented in Fig 2A.

Of the cases in which deaths occurred, 12 (63.2%) were associated with vascular access management problems, two each involved underlying diseases, unexpected cardiac events, and infection following complications, and one case involved the delay of dialysis treatment. The courts recognized malpractice in seven (58.3%) cases involving deaths related to vascular access problems, but in only two of the cases involving deaths of other causes (Fig 2B). Detailed information on the clinical situations for the malpractice cases is presented in Table 3. Cases that were dismissed are listed in S2 Table.

**Table 3:** Summary of general information of the lawsuits which recognized negligence.

No.	Age	Sex	Year of event	Type of kidney disease	Complications	Admitted violations of	Grave injury	Claimed amounts, USD	Trial outcome
						duty by judgment			
1	34	M	1991	CKD	Arterial rupture after dialysis catheter insertion	Malpractice during procedure	Death	58,333	Partial recognition
2	50	M	1998	AKI	Pseudoaneurysm after dialysis catheter insertion	Improper instruction and explanation	Nerve injury	32,026	Partial recognition
3	46	F	1998	CKD	Delayed dialysis due to delayed insertion of dialysis catheter	Delayed treatment and inappropriate management of complications.	Death	108,933	Conciliation
4	NI	M	1999	CKD	Nerve injury after access formation	Malpractice during surgery	Paralysis of upper arm	20,122	Partial recognition
5	NI	NI	2000	AKI	Infection related to multiple failure of dialysis catheter insertion	Malpractice during procedure and improper management of complication	Death	16,667	Conciliation
6	NI	M	2002	AKI	Respiratory distress due to hematoma after dialysis catheter insertion	Malpractice during procedure and improper management of complication	Death	115,894	Settlement decision
7	NI	M	2003	CKD	HCV infection associated with dialysis machine	Negligence of infection control in dialysis room	HCV infection	41,578	Partial recognition
8	NI	F	2005	CKD	Air embolism after dialysis catheter removal	Insufficient risk notification, prevention efforts, and management of complications	Death	227,582	Partial recognition
9	67	M	2007	CKD	HCV infection associated with dialysis machine	Negligence of infection control in dialysis room	HCV infection	243,642	Partial recognition
10	NI	NI	2008	CKD	Bone transformation due to exacerbation of hyperparathyroidism	Negligence in management of complication	Leg deformity	16,667	Partial recognition
11	68	F	2010	CKD	Nerve injury during surgery for AVF aneurysm following epidural anesthesia	Delayed diagnosis and treatment of complication	Paraplegia	360,635	Partial recognition
12	16	M	2011	CKD	Bleeding at the dialysis catheter insertion site	Insufficient management of complication	Death	36,324	Conciliation
13	NI	M	2012	CKD	Fall down after dialysis	Negligence in duty of care related to patient safety	Death	29,332	Partial recognition
14	NI	F	2014	CKD	Vascular obstruction distal to vascular access	Delayed diagnosis of complication	Amputation of fingers	27,245	Partial recognition
15	39	F	2014	CKD	Respiratory distress during access formation on epidural anesthesia	Malpractice during insertion of epidural catheter	Death	320,983	Partial recognition
16	NI	NI	2016	CKD	Delayed dialysis due to refuse of treatment by doctor	Improper instruction and explanation	None	5,417	Conciliation
17	32	M	2016	CKD	Burn by infrared warmer during dialysis	Violation in duty of care during dialysis and insufficient management of complication	3rd degree burn, bone destruction	348,493	Partial recognition
18	NI	M	2016	CKD	Bleeding after removal of dialysis needle from AVF	Delayed detection of complication	Death	47,270	Partial recognition

Abbreviations: AKI = Acute kidney injury; AVF = Arteriovenous fistula; CKD = chronic kidney disease; F = female sex; HCV = hepatitis C virus; M = Male; NI = Not identifiable; USD = United States Dollar; the exchange rate was 1 United States Dollar (USD) = 1200 Korean Won (KRW)

Following three cases were selected as representative cases what the court decided as negligence of the specific duty of care. For each case, we provide a summary, recognized negligence, and the reason for the limitation of liability.

Case 1. A 50-year-old male was diagnosed with acute kidney injury and had to undergo emergency dialysis treatment. Although it was stated at the permission obtained from patient’s family for the procedure as a dialysis catheter would be placed to right femoral vein, but an access through right subclavian vein was attempted. During the catheterization, right subclavian artery was punctured by mistake, but at first it seemed to be controlled by manual compression. After dialysis treatment, the patient complained right shoulder pain and paralysis of the right arm. Later, a pseudoaneurysm of the right subclavian artery was found, which damaged the brachial plexus. Pain and paralysis remained despite a surgical treatment to remove the pseudoaneurysm was done. The plaintiff sued for violating the duty of explanation and care, and the court recognized improper performance in catheterization and the negligence of the duty to explain.

Case 2. A woman of unknown age started hemodialysis treatment for chronic kidney disease. Although it is recommended that the double lumen catheter for hemodialysis should be removed from the supine position, but it was performed in the sitting position. Immediately after the procedure, an air embolism was developed and the patient died. The plaintiff claimed that the procedure was performed without proper notice and treatment. The court admitted that treatment was insufficient and inadequate, and risk notification and prevention efforts were also insufficient.

Case 3. A man of unknown age was diagnosed with acute kidney injury due to contrast induced nephropathy after coronary angiography, and it was decided to initiate hemodialysis treatment. A double lumen catheter was inserted into the left subclavian vein, because an access to the right internal jugular vein was tried, but was not successful. One hour after the procedure, a hematoma was found in the right neck at the catheterization trial site, and the bleeding was not properly controlled even after manual compression. Five hours after the procedure, the patient complained of dyspnea, and respiratory arrest occurred. The patient reached a coma with hypoxic brain damage, and eventually died due to septic shock. The plaintiff claimed errors in catheterization procedure, insufficient treatment for bleeding after the procedure, insufficient prophylactic treatment for dyspnea, inadequate monitoring for dyspnea, and the court admitted everything.

## Discussion

The present study reviewed 32 cases of medical malpractice related to hemodialysis procedures and vascular access management that were tried in the Korean court system. Most disputes were associated with events that occurred before or after, rather than during hemodialysis treatment, and associated mainly with vascular access placement and management. In addition, the majority of these complications resulted in serious clinical consequences.

Despite the great value of hemodialysis for controlling of uremia, the characteristics of patients with chronic kidney disease, who have multiple comorbidities and exposed to repeated hemodialysis procedures over long time period, increased the risk of complications even in cases when the procedures are performed by specialized medical staff [17–19]. This study showed that legal disputes related to hemodialysis treatment and vascular access management can be brought against any type of medical institution, regardless of the level of the institution (primary, secondary, or tertiary), or against both nursing and medical staff performing hemodialysis. The heterogeneity of defendants and event locations may reflect the complexity of hemodialysis procedures.

In the current study, the most common cause of disputes was vascular access management–related complications. In a recent study of medical dispute cases tried between 2005 and 2014 in the United States and recorded in the WESTLAW database [13], 66 cases involved complications arising from vascular access establishment or management (e.g., double-lumen CVC insertion or AVF creation/graft placement). Considering the annual incidence of hemodialysis treatment in Korea [20], which is about one-tenth that in the United States, the rate at which such legal disputes have been tried in Korea (12 cases over 19 years) is similar to that in the United States. In addition, the rate of litigations related to complications occurring during CVC insertion (25.0%) was greater than those due to complications related to AV access cannulation (6.3%) and formation (21.9%). Similar results were obtained in a study of malpractice suits brought on behalf of patients undergoing hemodialysis in the United States, in which the rate of CVC-associated cases (36%) was higher than those of cases associated with other types of vascular access (AVF formation, 18%; AV grafting, 12%) [13].

**Fig 3:**
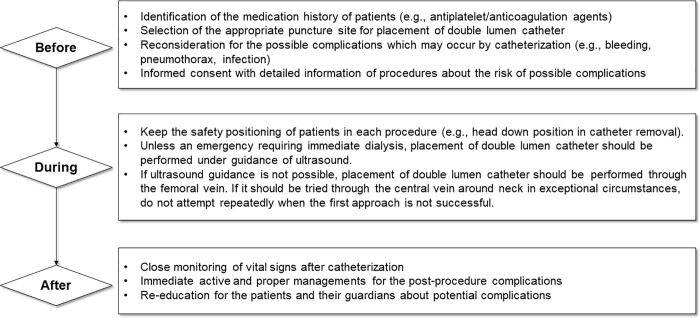
Recommendations for the prevention of malpractices during hemodialysis catheter-related procedures.

Medical staff caring for patients undergoing hemodialysis should be aware of the high rate of CVC-associated malpractice cases, as CVC insertion remains the most commonly performed procedure upon hemodialysis treatment initiation. According to the annual report of the United States Renal Data System, hemodialysis treatment is initiated by CVC for 80.3% of patients with incident kidney failure [1]. Although ‘fistula first’ strategy was recommended and the long-term CVC use was discouraged by the previous Kidney Disease Outcomes Quality Initiative vascular access guideline [21], the newer guideline recognized that CVCs may be appropriate for certain circumstances, such as in an older hemodialysis patients with limited life expectance or those with poor vascular access sites [22]. With this change in strategy for vascular access considering end-stage kidney disease life-plan and the increase of proportion of elderly dialysis patients, the high prevalence of CVC insertion for initial vascular access has hardly changed for 10 years [23, 24]. Nevertheless, current guidelines for the management of vascular access for dialysis in the United States [21, 22], Australia [25], and Europe [26] do not clearly define or describe all potential risks associated with CVC insertion, such as severe bleeding and air embolism; they describe only the risk of infection. In contrast, they provide detailed recommendations for the avoidance of complications associated with AV access cannulation [22]. According to the results of our study, most of the complications related to CVC was occurred during the insertion process, and it was presumed to be occurred while being performed by a trainee in an emergency situation. This could be a matter of skillfulness in the person inserting catheter, but it also should be emphasized that the procedure may have been performed without awareness of the safety process during catheterization. Based on the cases identified in our study and clinical experience, we suggested a framework that may be helpful to prevent errors during the procedure associated with implementation of dialysis catheter (Fig 3). By recognizing risks in each step of procedure clearly, the occurrences of serious and undesirable medical errors are supposed to be minimized. Ultimately, specific guidelines for CVC-related procedures should be established as well as the awareness for the risk of procedure-related complications in all medical staff should be raised to protect patients from procedure-related errors.

This study has several limitations. First, as complete medical records with precise descriptions of clinical situations (e.g., causes of kidney failure, comorbidities, vital signs, and laboratory findings) were not available, the claim-generating events may have had other causes. Second, the final judgment was based primarily on legal considerations, and were not made by medical specialists. Lastly, few medical litigation cases were brought during the 20-year study period. However, as the number of patients receiving dialysis in Korea is increasing rapidly (S3 Table), the number of dialysis-related disputes is expected to increase exponentially in the near future. In this regard, the present findings provide actual examples of the dialysis-related disputes, which may give a caution to medical staffs in dialysis units for avoiding recurrence of malpractices. And our finding demonstrates that medical staffs need to be alert when providing dialysis-related patient care.

In conclusion, hemodialysis -related legal cases tried during the past 19 years were analyzed in the present study. Given the severity of the complications as well as the characteristics of malpractice which could be prevented by increased efforts of medical staffs, it is important to raise the attention in malpractice prevention by medical staffs dedicated to hemodialysis treatment with thorough review of previous cases. Awareness of existing guideline recommendations for these procedures, and close monitoring of patients’ conditions throughout the hemodialysis process, are essential to reduce the risk of malpractice. Better outcomes for patients undergoing hemodialysis, with increased safety and a reduced risk of litigation, can be achieved through the efforts of all medical staff involved in hemodialysis.

## Supporting information

S1 Table Judicial characteristics of the litigation cases. (DOCX)

S2 Table Detailed summary of dismissed fourteen-cases concerning hemodialysis lawsuits. (DOCX)

S3 Table Number of hemodialysis patients and sessions and total cost of hemodialysis in Korea over the past five years. (DOCX)

## References

[1] Saran R, Robinson B, Abbott KC, Agodoa LYC, Bragg-Gresham J, Balkrishnan R, et al. US Renal Data System 2018 Annual Data Report: Epidemiology of Kidney Disease in the United States. Am J Kidney Dis. 2019;73(3 Suppl 1):A7–A8. Epub 2019/02/26. doi: 10.1053/j.ajkd.2019.01.001 ; PubMed Central PMCID: PMC6620109. 30798791 PMC6620109

[2] Himmelfarb J. Hemodialysis complications. Am J Kidney Dis. 2005;45(6):1122–31. Epub 2005/06/16. doi: 10.1053/j.ajkd.2005.02.031 . 15957144

[3] Grebenyuk LA, Marcus RJ, Nahum E, Spero J, Srinivasa NS, McGill RL. Pulmonary embolism following successful thrombectomy of an arteriovenous dialysis fistula. J Vasc Access. 2009;10(1):59–61. Epub 2009/04/03. doi: 10.1177/112972980901000111 . 19340802

[4] Karnik JA, Young BS, Lew NL, Herget M, Dubinsky C, Lazarus JM, et al. Cardiac arrest and sudden death in dialysis units. Kidney Int. 2001;60(1):350–7. Epub 2001/06/26. doi: 10.1046/j.1523-1755.2001.00806.x . 11422771

[5] Munger MA, Ateshkadi A, Cheung AK, Flaharty KK, Stoddard GJ, Marshall EH. Cardiopulmonary events during hemodialysis: effects of dialysis membranes and dialysate buffers. Am J Kidney Dis. 2000;36(1):130–9. Epub 2000/06/30. doi: 10.1053/ajkd.2000.8285 . 10873882

[6] Skroeder NR, Jacobson SH, Lins LE, Kjellstrand CM. Acute symptoms during and between hemodialysis: the relative role of speed, duration, and biocompatibility of dialysis. Artif Organs. 1994;18(12):880–7. Epub 1994/12/01. doi: 10.1111/j.1525-1594.1994.tb03339.x . 7887824

[7] Prabhakar Singh RG, Singh S, Rathore SS, Choudhary TA. Spectrum of intradialytic complications during hemodialysis and its management: a single-center experience. Saudi J Kidney Dis Transpl. 2015;26(1):168–72. Epub 2015/01/13. doi: 10.4103/1319-2442.148771 . 25579743

[8] Baldwin JJ, Edwards JE. Uremic pericarditis as a cause of cardiac tamponade. Circulation. 1976;53(5):896–901. Epub 1976/05/01. doi: 10.1161/01.cir.53.5.896 . 1260996

[9] Fraser CL, Arieff AI. Nervous system complications in uremia. Ann Intern Med. 1988;109(2):143–53. Epub 1988/07/15. doi: 10.7326/0003-4819-109-2-143 . 2837930

[10] Albers FJ. Causes of hemodialysis access failure. Adv Ren Replace Ther. 1994;1(2):107–18. Epub 1994/07/01. doi: 10.1016/s1073-4449(12)80042-2 . 7614311

[11] Nassar GM, Ayus JC. Infectious complications of the hemodialysis access. Kidney Int. 2001;60(1):1–13. Epub 2001/06/26. doi: 10.1046/j.1523-1755.2001.00765.x . 11422731

[12] Wang K, Wang P, Liang X, Lu X, Liu Z. Epidemiology of haemodialysis catheter complications: a survey of 865 dialysis patients from 14 haemodialysis centres in Henan province in China. BMJ Open. 2015;5(11):e007136. Epub 2015/11/22. doi: 10.1136/bmjopen-2014-007136 ; PubMed Central PMCID: PMC4663418. 26589425 PMC4663418

[13] Phair J, Carnevale M, Wilson E, Koleilat I. Jury verdicts and outcomes of malpractice cases involving arteriovenous hemodialysis access. J Vasc Access. 2019:1129729819872846. Epub 2019/09/10. doi: 10.1177/1129729819872846 . 31495258

[14] Miller LM, MacRae JM, Kiaii M, Clark E, Dipchand C, Kappel J, et al. Hemodialysis Tunneled Catheter Noninfectious Complications. Can J Kidney Health Dis. 2016;3:2054358116669130. Epub 2017/03/09. PubMed Central PMCID: PMC5332086. doi: 10.1177/2054358116669130 28270922 PMC5332086

[15] Cho SI, Shin SH, Yang JH, Lee W, Kim SY, Suh DH. Analysis of acne-related judicial precedents from 1997 to 2018 in South Korea. J Dermatol. 2019;46(12):1210–4. Epub 2019/10/24. doi: 10.1111/1346-8138.15119 . 31642108

[16] Shin SH, Kim SY, Jang SG, Lee W. Analysis of closed medical litigation in urology. Investig Clin Urol. 2017;58(5):317–23. Epub 2017/09/05. doi: 10.4111/icu.2017.58.5.317 ; PubMed Central PMCID: PMC5577327. 28868502 PMC5577327

[17] Chao CT, Huang JW, Yen CJ. Intradialytic hypotension and cardiac remodeling: a vicious cycle. Biomed Res Int. 2015;2015:724147. Epub 2015/02/06. doi: 10.1155/2015/724147 ; PubMed Central PMCID: PMC4310253. 25654122 PMC4310253

[18] Rayner HC, Zepel L, Fuller DS, Morgenstern H, Karaboyas A, Culleton BF, et al. Recovery time, quality of life, and mortality in hemodialysis patients: the Dialysis Outcomes and Practice Patterns Study (DOPPS). Am J Kidney Dis. 2014;64(1):86–94. Epub 2014/02/18. doi: 10.1053/j.ajkd.2014.01.014 ; PubMed Central PMCID: PMC4069238. 24529994 PMC4069238

[19] Morfin JA, Fluck RJ, Weinhandl ED, Kansal S, McCullough PA, Komenda P. Intensive Hemodialysis and Treatment Complications and Tolerability. Am J Kidney Dis. 2016;68(5S1):S43–S50. Epub 2016/10/25. doi: 10.1053/j.ajkd.2016.05.021 . 27772642

[20] Jin DC, Yun SR, Lee SW, Han SW, Kim W, Park J, et al. Current characteristics of dialysis therapy in Korea: 2016 registry data focusing on diabetic patients. Kidney Res Clin Pract. 2018;37(1):20–9. Epub 2018/04/10. doi: 10.23876/j.krcp.2018.37.1.20 ; PubMed Central PMCID: PMC5875573. 29629274 PMC5875573

[21] Vascular Access Work G. Clinical practice guidelines for vascular access. Am J Kidney Dis. 2006;48 Suppl 1:S248–73. Epub 2006/07/04. doi: 10.1053/j.ajkd.2006.04.040 . 16813991

[22] Charmaine E. Lok TSH, Timmy Lee, Surendra Shenoy, Alexander S. Yevzlin, Kenneth Abreo, Michael Allon AA, et al. KDOQI Vascular Access Guideline Work Group. KDOQI clinical practice guideline for vascular access: 2019 update. Am J Kidney Dis. 2020;75(4)(suppl 2):S1–S164. 32778223 10.1053/j.ajkd.2019.12.001

[23] Johansen KL, Chertow GM, Foley RN, Gilbertson DT, Herzog CA, Ishani A, et al. US Renal Data System 2020 Annual Data Report: Epidemiology of Kidney Disease in the United States. Am J Kidney Dis. 2021;77(4 Suppl 1):A7–A8. Epub 2021/03/24. doi: 10.1053/j.ajkd.2021.01.002 ; PubMed Central PMCID: PMC8148988. 33752804 PMC8148988

[24] Lee HS, Song YR, Kim JK, Joo N, Kim C, Kim HJ, et al. Outcomes of vascular access in hemodialysis patients: Analysis based on the Korean National Health Insurance Database from 2008 to 2016. Kidney research and clinical practice. 2019;38(3):391–8. doi: 10.23876/j.krcp.19.015 ; PubMed Central PMCID: PMC6727887. 31378011 PMC6727887

[25] Polkinghorne KR, Chin GK, MacGinley RJ, Owen AR, Russell C, Talaulikar GS, et al. KHA-CARI Guideline: vascular access—central venous catheters, arteriovenous fistulae and arteriovenous grafts. Nephrology (Carlton). 2013;18(11):701–5. Epub 2013/07/17. doi: 10.1111/nep.12132 . 23855977

[26] Schmidli J, Widmer MK, Basile C, de Donato G, Gallieni M, Gibbons CP, et al. Editor’s Choice—Vascular Access: 2018 Clinical Practice Guidelines of the European Society for Vascular Surgery (ESVS). Eur J Vasc Endovasc Surg. 2018;55(6):757–818. Epub 2018/05/08. doi: 10.1016/j.ejvs.2018.02.001 . 29730128

