# Supplementary material for: Medical malpractice related to dialysis and vascular access: An analysis of lawsuit judgements in South Korea
Source: PLoS One. 2021 Aug 5;16(8):e0255020. doi: 10.1371/journal.pone.0255020 (PMC8341505; doi:10.1371/journal.pone.0255020)
Supplement: S1 Table — (DOCX) [file pone.0255020.s001.docx]

**S1 Table.** Judicial characteristics of the litigation cases

| Characteristics | N=32 |
| --- | --- |
| Type of defendant institution |  |
| Primary hospital | 8 (25.0%) |
| General hospital | 10 (31.3%) |
| Tertiary hospital | 14 (43.8%) |
| Progress of lawsuit |  |
| District Court | 19 (59.4%) |
| Appeal | 11 (34.4%) |
| Final appeal | 2 (6.3%) |
| Trial outcome |  |
| Dismissal | 13 (40.6%) |
| Conciliation or Settlement decision | 6 (18.7%) |
| In favor of plaintiff | 13 (40.6%) |
| Awarded to plaintiff | 59.40% |
| Defendant |  |
| Nurse | 3 (9.4%) |
| Doctor | 10 (31.3%) |
| Hospital | 24 (75.0%) |
| Amount for claim – median USD (IQR) | 72,506 (27,144-262,977) |
| Awarded amount for damage – median USD (IQR) | 21,667 (9,583-73,807) |

Data are presented as number (%), median (interquartile range).

USD = United States Dollar; the exchange rate was 1 United States Dollar (USD)=1200 Korean Won (KRW), IQR = interquartile range.
