# Supplementary material for: Medical malpractice related to dialysis and vascular access: An analysis of lawsuit judgements in South Korea
Source: PLoS One. 2021 Aug 5;16(8):e0255020. doi: 10.1371/journal.pone.0255020 (PMC8341505; doi:10.1371/journal.pone.0255020)
Supplement: S2 Table — (DOCX) [file pone.0255020.s002.docx]

**S2 Table**. Detailed summary of dismissed fourteen-cases concerning hemodialysis lawsuits.

| **No.** | **Year** | **Reason for dialysis** | **Final state** | **Allegation of plaintiff** | **Judgment by the court (reasons for dismissal)** |
| --- | --- | --- | --- | --- | --- |
| 1 | 2017 | AKI | death | Patient died from pneumonia after autologous stem cell transplantation for multiple myeloma treatment. The plaintiff alleged violation of standard precaution for infection during dialysis. | No proven negligence |
| 2 | 2015 | CKD | recovered | Heparin was not administered during dialysis, leading to blood clotting and stopping dialysis. | No proven negligence |
| 3 | 2014 | CKD | death | In a primary clinic, the dialyzer accidentally stopped due to a power outage during dialysis, leading to patient's death. | No proven negligence. It is not a legal obligation to install an emergency power supply in primary clinics. |
| 4 | 2011 | CKD | unknown | Bacterial infection occurred during dialysis due to the fault in arteriovenous access needle insertion. | No proven negligence. There was already an evidence of infection before dialysis. |
| 5 | 2007 | CKD | death | The patient had cardiac arrest after dialysis start with nurse while the doctor had not yet been to work. The plaintiff alleged that the patient had been treated without the supervision of a doctor. | Dialysis in the defendant's clinic has been done in advance by a doctor's instructions and in a pre-planned schedule and method. There is no 6evidence to admit the negligence of the d7efendant's direction and supervision. |
| 6 | 2004 | CKD | death | Subcutaneous swelling after intravenous injection during dialysis caused skin necrosis. The Plaintiff alleged negligence in duty of care and explanation for subcutaneous swelling. | No8 proven negligence. |
| 7 | 2006 | AKI | death | Patients with chronic kidney disease, who were hospitalized for recurrence of acute myeloid leukemia, stopped dialysis due to a dialysis machine failure, resulting the patient's general condition worsened. | No p9roven negligence. Hemodialysis was resumed within a few hours, and the temporary discontinuation of dialysis was not presumed to be the cause of the deterioration of the patient's condition. |
| 8 | 1994 | AKI | death | Hemothorax, which leading to pyothorax, was caused by a failed attempt to insert a hemodialysis catheter into the left subclavian vein. | No proven negligence. |
| 9 | 2012 | CKD | death | Hemothorax was caused by a failed attempt to insert a hemodialysis catheter due to unreasonable attempts by an inexperienced doctor. | The defendant is a general surgeon, qualified for the procedure, and there is no evidence to recognize medical negligence in treatment. |
| 10 | 2011 | CKD | death | Vascular rupture occurred due to negligence of attention during AV access needle insertion, resulting in skin necrosis. | No proven negligence. |
| 11 | 2015 | CKD | death | After AVF surgery, respiratory distress and cardiac arrest occurred, and the defendant did not fulfill the caution and explanation duties related to the surgery. | No proven negligence. |
| 12 | 2015 | CKD | hemiplegia | Cerebellar infarct occurred during heparin administration for splenic infarct and AVF obstruction, and the defendant had negligence of delayed diagnosis of brain hemorrhage and delayed suspension of heparin administration. | No proven negligence. |
| 13 | 2016 | CKD | Death | Patients died of pneumonia after AVF surgery was performed under general anesthesia, and the defendant had negligence in delayed diagnosis of pneumonia and the violation of explanation for general anesthesia. | The patient agreed on the possibility of changing the anesthesia method, and there is no evidence to recognize the negligence of delay in diagnosis. |
| 14 | 2008 | CKD | recovered | AVF obstruction occurred due to fault in compression of AVF after dialysis, resulting reoperation of AVF. | No proven negligence. |

Abbreviations: AKI, acute kidney injury; CKD, chronic kidney disease; AVF, arteriovenous fistula.
