# Supplementary material for: Medical malpractice related to dialysis and vascular access: An analysis of lawsuit judgements in South Korea
Source: PLoS One. 2021 Aug 5;16(8):e0255020. doi: 10.1371/journal.pone.0255020 (PMC8341505; doi:10.1371/journal.pone.0255020)
Supplement: S3 Table — (DOCX) [file pone.0255020.s003.docx]

**S3 Table.** Number of hemodialysis patients and sessions and total cost of hemodialysis in Korea over the past five years

| Years | Number of hemodialysis patients | Number of dialysis session | Total cost for hemodialysis (won) |
| --- | --- | --- | --- |
| 2015 | 57,622 | 6,438,465 | 597,610,307 |
| 2016 | 60,937 | 6,794,752 | 644,254,558 |
| 2017 | 63,625 | 7,167,604 | 695,154,922 |
| 2018 | 68,304 | 7,664,461 | 757,258,311 |
| 2019 | 71,568 | 8,128,156 | 817,020,604 |

This data was extracted from the Healthcare Big Data Hub of the Health Insurance Review & Assessment Service (HIRA) in Korea (http://opendata.hira.or.kr)
